# Severe exfoliative dermatitis induced by apalutamide in a mHSPC patient: A case report

**DOI:** 10.1097/MD.0000000000047428

**Published:** 2026-02-13

**Authors:** Hanzhong Chen, Teng Li, Junhong Fan, Jiumin Liu

**Affiliations:** a Department of Urology, Guangdong Provincial People’s Hospital (Guangdong Academy of Medical Sciences), Southern Medical University, Guangzhou, China.

**Keywords:** adverse reaction, apalutamide, exfoliative dermatitis, prostate cancer

## Abstract

**Rationale:**

Apalutamide is a generation androgen antagonist for the treatment of metastatic hormone sensitive prostate cancer and non-metastatic castration resistant prostate cancer. Skin adverse reactions caused by apalutamide are mostly rashes and maculopapules.

**Patient concerns:**

There are a few reports about exfoliative dermatitis caused by apalutamide.

**Diagnoses:**

Severe exfoliative dermatitis induced by apalutamide.

**Interventions:**

Gamma globulin, cefoperazone sulbactam sodium, albumin, ebastine, loratadine, hydrocortisone cream, and 3% boric acid lotion.

**Outcomes:**

Whole-body skin was red, without exudation, and with a small amount of desquamation.

**Lessons:**

The incidence of exfoliative dermatitis due to apalutamide is low but severe, and if a serious adverse skin reaction is suspected, apalutamide needs to be discontinued immediately and treated accordingly.

## 1. Introduction

Prostate cancer is among the most common cancers worldwide, with approximately 2.6 million new cases each year,^[1]^ novel insights and therapeutic approaches related to the genitourinary microbiome in prostate cancer patients are emerging.^[2]^ Apalutamide is a synthetic biaryl thiohydantoin compound and a new generation androgen antagonist for the treatment of prostate cancer. It can directly bind to the ligand-binding domain of the androgen-receptor, inhibit the nuclear transport of the androgen-receptor, and prevent androgen-receptor-mediated transcription, thus suppressing tumor growth. In phase III clinical trials, apalutamide treatment has shown an extension of metastasis-free survival and overall survival, and it is now 1 of the first-line drugs for the treatment of metastatic hormone sensitive prostate cancer (mHSPC) and non-metastatic castration resistant prostate cancer.^[3]^ Another study collects data for patients with prostate cancer treated in multiple centers worldwide to assess outcomes in the real-world setting, and results show that apalutamide is a safe and effective drug in the real-world setting as well as in clinical trials.^[4]^

Skin adverse reactions caused by apalutamide are mostly rashes and maculopapules.^[3]^ In addition, relatively rare cutaneous adverse events caused by apalutamide include erythema multiforme, urticaria, stomatitis, toxic epidermal necrolysis (TEN), etc.^[5]^ Till now, there are few reports about exfoliative dermatitis caused by apalutamide.(Table 1)^[6–12]^

**Table 1:** Reported patients with exfoliative dermatitis caused by apalutamide.

No.	Yr	Age (yr)	Dose (mg)	Duration (wk)	Type of skin eruption	Treatment	Outcome
1	2020	83	240	6	TEN	SC, IVIG, PA	Death
2	2020	77	240	2	TEN	SP, IVIG	Death
4	2021	85	N/A	5	DRESS syndrome	SC	Healed
5	2022	85	240	7	TEN	SC, PA	Death
6	2022	86	240	3.3	TEN	OS, SC, IVIG, PA	Healed
7	2022	91	240	7	TEN	SC, IVIG	Death
8	2022	77	240	6.1	TEN	SP, IVIG, cyclosporin	Healed
9	2023	77	240	5.7	SJS	SC,IVIG, TNF-a inhibitor	Healed
10	2023	69	240	16	SJS	SC, OS	Healed
11	2023	72	240	6.4	SJS	SC	Healed
12	2023	74	240	N/A	TEN	SC, adalimumab	Death

DRESS = drug reaction with eosinophilia and systemic symptoms, IVIG = intravenous immunoglobulin, NA = not applicable, OS = oral steroid, PA = plasmapheresis, SC = systemic corticosteroid, SP = steroid pulse, TEN = toxic epidermal necrolysis.

This article reports a case of severe exfoliative dermatitis induced by apalutamide in a Chinese mHSPC patient. Analysis of the patient was approved by the Medical Ethics Committee of Guangdong Provincial People’s Hospital. Informed consent was obtained from the patient for the publication of this case report details.

## 2. Clinical data

**Figure 1.**
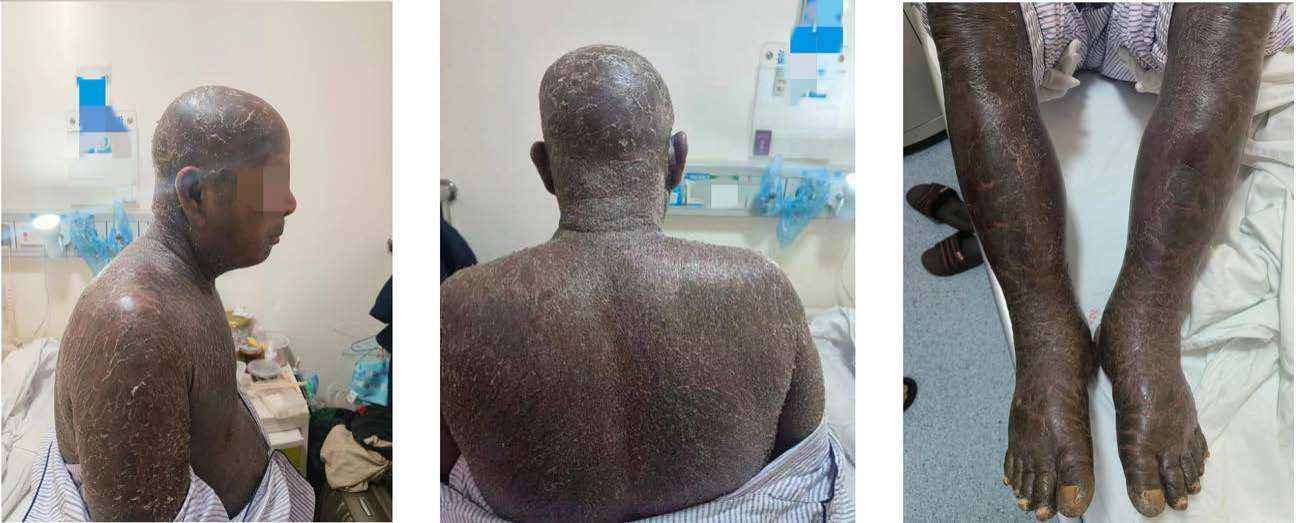
Before admission, the patient’s whole-body skin showed exudation and large-scale desquamation.

**Figure 2.**
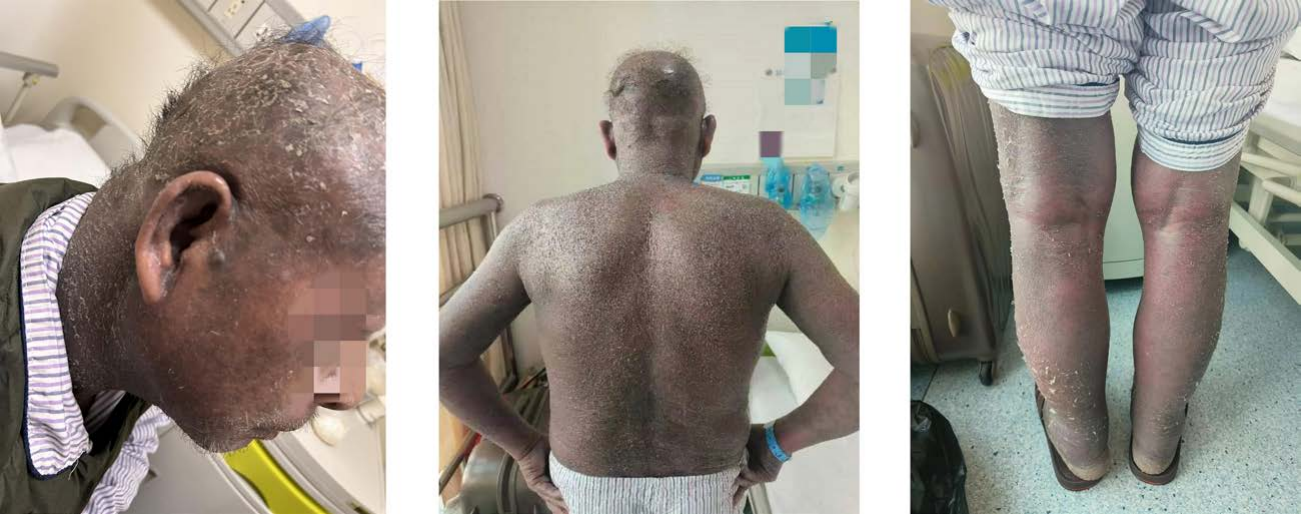
After 7 days of treatment, the patient’s symptoms started to relieve.

**Figure 3.**
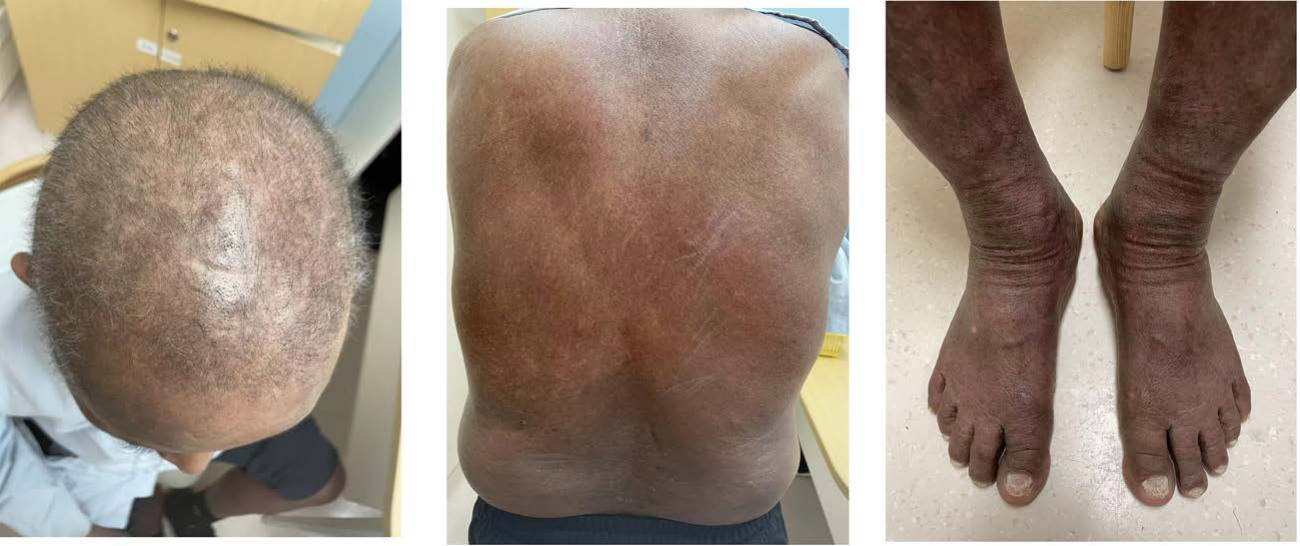
After 2 months of treatment, the whole-body skin was red, without exudation, and with a small amount of desquamation.

The patient, a 73-year-old male, was admitted to our hospital due to “skin redness generalisatus for more than 3 months, aggravated for 1 month.” The patient was diagnosed with mHSPC, 14 days after taking apalutamide (240 mg/d), the rash began on the abdomen and upper limbs and gradually spread throughout the body, accompanied by itching. The patient was asked to stop taking apalutamide, but the symptoms still worsened. The patient had visited the hospital several times, and allergic dermatitis was considered. After treatment with mometasone furoate, there was no improvement. 2 months later, the patient’s whole-body skin gradually showed exudation and large-scale desquamation (Fig. 1), accompany with slight fever. The patient visited the clinic, and exfoliative dermatitis (drug eruption caused by apalutamide) was considered; further hospitalization was required. The patient has no other medical conditions. On admission, physical examination showed: body temperature (T) 39.0°C, the skin on the face, neck, trunk, and limbs was diffusely flushed and swollen, with scaly desquamation all over the body. The epidermis at the skin-mucosa junction was eroded and exuded, the conjunctiva was edematous, and the superficial lymph nodes all over the body were not enlarged. The results of cardiopulmonary and abdominal examinations were negative (−). During hospitalization, blood test results were as follows: white blood cell 11.03 × 10^9^ L^−1^; neutrophil percentage 51.1%; eosinophil percentage 22.4%; albumin 29 g L^−1^; interleukin-6 42.8 pg/mL; and the rest were normal. On the day of admission, gamma globulin was used to enhance immunity (20 g qd for 5 days), cefoperazone sulbactam sodium (3 g, twice a day) was given for anti-inflammation, albumin was provided for nutritional support, ebastine and loratadine were used for anti-allergy, hydrocortisone cream was applied externally, and 3% boric acid lotion was used for wet dressing. After 7 days of treatment, the patient’s symptoms started to relieve (Fig. 2). 2 months later, physical examination showed that the whole-body skin was red, without exudation, and with a small amount of desquamation (Fig. 3).

Overall, the severity of the skin lesions exceeded the expectations of the patient and their family. If they had been informed in advance of the possible most severe conditions, they would have paid more attention to the skin problem, thereby discontinuing the medication earlier, and may ultimately achieve a better prognosis.

## 3. Discussion

This case report alerts urologists and patients to pay extra attention to skin reactions as an adverse reaction when using apalutamide. According to 2 studies (global SPARTAN and TITAN studies), the incidence of skin rashes in the apalutamide group was 23.8% and 27.1%, respectively.^[13]^ In contrast to apalutamide, other nonsteroidal androgen-receptor inhibitors, such as enzalutamide, are not commonly associated with a high incidence of skin rash. The pathological mechanism of skin adverse reactions caused by apalutamide is still unclear. The increased incidence of apalutamide-related rashes may be related to structural differences, the 2-cyanopyridine moiety in apalutamide can react with cysteine residues in serum proteins to form a hapten-protein complex, which triggers an immune response and increases the likelihood of rashes.^[14]^

While Yoichiro Tohi et al suggested that the apalutamide-associated skin rash might not be attributed to allergic reactions but rather to a structure-specific, off-target pharmacological reaction.^[15]^ Additionally, low body weight may be a risk factor for apalutamide-related cutaneous adverse events.^[16]^ In this case, the exfoliative dermatitis caused by apalutamide belongs to a severe drug eruption. The clinical manifestations include symptoms such as diffuse skin redness, erosion, exudation, and desquamation. In patients with heart failure, sepsis, and pneumonia may even cause death. The pathogenesis of exfoliative dermatitis is still unclear. It may be due to the complex interaction between adhesion molecules in epithelial cells and drugs, which leads to a large number of inflammatory cells aggregating in the skin. This affects the time, speed, or renewal and metabolism level of epidermal cell mitosis, resulting in skin exfoliation.^[17]^

In cases of TEN caused by apalutamide, the time interval for patients to develop rashes after taking apalutamide seems to be shorter (2–6 weeks) than in other cases.^[18]^ The early appearance of rashes in patients taking apalutamide may be a sign of severe disease. In this case, the patient developed local skin redness 2 weeks after taking apalutamide, and the symptoms continued to worsen even after the drug was discontinued, which may be related to the patient’s advanced age and poor body metabolism. Coupled with the patient’s advanced age and previous prostate cancer, the body’s resistance is weak. Once an infection occurs, it is likely to develop into a severe infection with a poor prognosis. The lethal rate associated with apalutamide-related TEN seems to be higher than that with other drugs (66.7% vs 19%–44%), and this poorer prognosis may be attributable to a relatively long metabolic period of apalutamide (half-life, 5–6 days).^[5]^ There are some limitations in this case report; the patient in this case did not undergo a pathological biopsy of the skin lesion, and the exact mechanism that causes such adverse reactions in this patient remains unclear. Further research should be conducted to clarify the mechanism of severe cutaneous adverse reactions caused by apalutamide and develop individualized treatment regimens.

Therefore, before choosing apalutamide to treat elderly patients with prostate cancer, they should be fully informed of its adverse reactions. If symptoms such as skin redness and exudation occur, they should seek medical attention in a timely manner for early treatment to prevent the aggravation of the condition. Overall, the incidence of exfoliative dermatitis due to apalutamide is low but severe, and if a serious adverse skin reaction is suspected, apalutamide needs to be discontinued immediately and treated accordingly.

## Acknowledgments

This work was supported by grants from the Guangdong Provincial Department of Finance Project in 2022 (KS0120220271, KS0120220272).

## Author contributions

**Data curation:** Hanzhong Chen, Teng Li, Junhong Fan.

**Writing–original draft:** Hanzhong Chen, Teng Li, Junhong Fan.

**Writing–review & editing:** Hanzhong Chen, Jiumin Liu.
